# Screening and Identification of B-Cell Epitopes in the P61 Protein of *Nocardia brasiliensis*

**DOI:** 10.3389/fcimb.2018.00224

**Published:** 2018-07-02

**Authors:** Xingzhao Ji, Na Li Sun, Xin Xue Hou, Shuai Xu, Tong Xiao Qiu, Lu Tang, Qiao He Li, Bing Xue Wang, Jun Zhen Li

**Affiliations:** ^1^State Key Laboratory for Infectious Disease Prevention and Control, Chinese Center for Disease Control and Prevention, National Institute for Communicable Disease Control and Prevention, Beijing, China; ^2^Key Laboratory of Laboratory Medicine, Ministry of Education, School of Laboratory Medicine and Life Sciences, Wenzhou Medical University, Wenzhou, China

**Keywords:** *Nocardia brasiliensis*, P61, monoclonal antibodies, B-cell epitope, serodiagnostic

## Abstract

The P61 protein is an immunodominant antigen of *Nocardia brasiliensis* that is observed in the sera from patients infected with the bacterium. However, the B-cell epitopes of *N. brasiliensis* are still unresolved. To identify the antigenic determinants of P61, we screened seven monoclonal antibodies (mAbs) against P61 protein that was expressed in the *Escherichia coli* system. A series of truncated peptides of P61 were then generated and the mAbs were used to screen these peptides by Western blot analyses. Three B-cell epitopes were recognized by the P61 specific mAbs: 461-FEYWTKVDPEIGKRIEEG-478, 427-LVREVFNDAQRDRLVSNVVGGVQEPV. LSRVFEYWTKVDPEIGKRIEEGVRAG-482, and 447-HVLGGVQEPVLSRVFEY WTKVDPEI GKRIEEGVRAGLD-484. The latter two epitopes were further identified by *N. brasiliensis*-infected mouse serum. These results facilitate future investigations of serodiagnostic methods to identify *Nocardia* infections.

## Introduction

*Nocardia brasiliensis* is a facultative, intracellular, filamentous, gram-positive, and partially acid-fast bacterium that infects skin via traumatic inoculation. Infection produces symptoms of chronic inflammation including fistulas, abscesses, cellulitis, ulcers, and mycetoma (Smego and Gallis, 1984; Salinas-Carmona, 2000; Salinas-Carmona et al., 2009). Infections can later spread to muscles, bones, and adjacent organs. Infections can also be transmitted via cutaneous or respiratory inhalation resulting in CNS disease (Smego and Gallis, 1984; Beaman and Beaman, 1994; Chen et al., 2016). Primary cutaneous nocardiosis is an infectious disease caused by bacteria in the genus *Nocardia*, but is most often caused by *N. brasiliensis* infection (Smego and Gallis, 1984; Wilson, 2012; Chen et al., 2016). *N. brasiliensis* is the primary etiologic agent of human mycetoma in Mexico, and about 86% of the mycetoma cases there are caused by the bacterium (Lopez Martinez et al., 1992; Salinas-Carmona et al., 1992; Licón-Trillo et al., 2003; Castro-Matteotti et al., 2008). The numbers of human nocardiosis cases are increasing in developed countries, and especially in immunocompromised patients (Salinas-Carmona et al., 1992; Brown-Elliott et al., 2006). The proper diagnosis of this disease is therefore important to promote rapid and efficient clinical treatment of infected patients. The diagnosis of mycetoma caused by *N. brasiliensis* is currently based on isolation and cultivation techniques. However, the confirmation of its presence using conventional microbiological techniques usually takes quite a long time, and it is also difficult to clinically differentiate *N. brasiliensis* infections from cutaneous infection by *Staphylococcus aureus* and *Streptococcus pyogenes* (Chen et al., 2016). Further, *N. brasiliensis* is closely related to *Mycobacterium tuberculosis* and shares many of its morphological, antigenic, and physiological characteristics (Castro-Matteotti et al., 2008). Due to a lack of purified antigens, the cross-reactivity of *N. brasiliensis* antigens with sera from leprosy and tuberculosis patients remains an important, unresolved problem in disease diagnosis (Humphreys et al., 1975; Salinas-Carmona et al., 1992).

Two proteins from a culture filtrate of *N. asteroides* with molecular weights of 55,000 and 31,000 Da have been demonstrated as highly specific markers to identify patients infected with *N. asteroides*, and can thus be used as diagnostic tools for nocardiosis (Sugar et al., 1985). In addition, Vera-Cabrera et al. purified two proteins from a crude extract of *N. brasiliensis*, termed P61 and P24. These two proteins are recognized by sera from nocardial mycetoma patients, but not sera from mycobacterial-infected or healthy controls, and therefore could have great value in the serological diagnosis of *N. brasiliensis-*infected patients (Vera-Cabrera et al., 1992). The P61 protein is encoded by the gene *O3I_001640* and is the target of the humoral immune response in patients suffering from nocardial mycetoma (Gordon et al., 2013). This immunodominant protein is highly conserved in the *Nocardia* genus (Vera-Cabrera et al., 1999), furthering its potential as a tool for clinical diagnosis of nocardiosis.

Clinical diagnosis assays relying on synthesized peptides are considered to have more advantages than those using recombinant or native protein antigens (Goyal et al., 2014). Consequently, it is important to analyze specific epitopes for the development of epitope peptide-based diagnostic tools. B-cell epitopes are regions on the surface of the native antigen that are recognized by binding to B-cell receptors or specific antibodies (Viudes et al., 2001; Zhang et al., 2015). To date, there has been no validation of a peptide-based serodiagnostic assay of the P61 protein. In this study, we generated seven monoclonal antibodies (mAbs) against recombinant P61 protein and used them to screen for B-cell epitopes using Western blot analyses. Two epitopes were additionally recognized by *N. brasiliensis*-infected mouse serum. These results represent a promising first step in the development of specific serological tools for nocardiosis diagnosis.

## Materials and methods

### Ethics statement

Laboratory animal care and experimentation were conducted in accordance with animal ethics guidelines and protocols approved by the Ethics Review Committee of the National Institute for Communicable Disease Control and Prevention at the Chinese Center for Disease Control and Prevention.

### Bacterial strains, plasmids, and animals

The standard strain of *N. brasiliensis* (ATCC700358) was purchased from the German Resource Centre for Biological Materials and grown in brain-heart-infusion (BHI) medium (Difco Laboratories, Detroit, MI), as previously described (Vera-Cabrera et al., 1992; Salinas-Carmona et al., 1999). pET30a and pMAL-c5x plasmids were used as expression vectors (New England Biolabs, Beijing) and *E. Coli* strain BL21 (DE3) was used as the vector host. *E. coli* (TransGen Biotech, China) was grown in Luria-Bertani (LB) medium. Female BALB/c mice that were 9–12 weeks of age were maintained under pathogen-free conditions and used for serological testing.

### Preparation of P61 protein

P61 protein that is used as an antigen in the generation of mAbs was expressed in BL21 (DE3) *E. coli* cells. Briefly, the katN gene codon was optimized and synthesized by Sangon Biotech (the restriction endonuclease sites are *NdeI* and *HindIII*), cloned into the expression vector pET-30a(+), and transformed into BL21 (DE3) competent cells. Recombinant BL21 cells were cultured at 37°C in LB medium containing 50 μg/ml kanamycin with agitation until their optical density (measured at 600 nm) reached 0.8. Expression was then induced with 0.2 mM isopropyl β-D-1-thiogalactopyranoside (IPTG) at 30°C for 6 h. Cells were sonicated and centrifuged at 12,000 rpm for 20 min at 4°C. The supernatant was then carefully collected. Production of recombinant P61 protein was evaluated by sodium dodecyl sulfate-polyacrylamide gel electrophoresis (SDS-PAGE). The expressed P61 proteins were purified with a Ni-NTA kit (Novagen) according to the manufacturer's instructions and stored at −80°C.

The purified, recombinant P61 protein was confirmed by Western blot analysis. Briefly, purified recombinant P61 and *N. brasiliensis* whole-cell protein were electro-blotted onto a polyvinylidene fluoride (PVDF) membrane at 100 mA for 1 h, then blocked overnight at 4°C in blocking buffer (5% skim milk in PBS, pH 7.4, with 0.05% Tween 20). Membranes were incubated at room temperature with anti-*N. brasiliensis* mouse serum for 2 h, and then incubated with HRP-conjugated goat anti-mouse IgG (TransGen Biotech, China) for 1 h. Protein detection was performed using chemiluminescent luminol reagents (Takara, China).

### Preparation and identification of mAbs against P61 protein

We used standard hybridoma techniques to screen for specific anti-p61 MAbs (Chaithirayanon et al., 2002). Briefly, purified His-P61 protein was emulsified with equal volumes of complete/incomplete Freund's adjuvant (Sigma–Aldrich) at a final concentration of 0.25 mg/ml. Female BALB/c mice (6–8 weeks old) were subcutaneously immunized with purified His-P61 protein in 2 week intervals over 6 weeks to generate hybridoma lines that secreted antibodies. The spleen cells of immunized BALB/c mice were fused with mouse myeloma cells (SP2/0) 3 days after the last injection. The fused hybridoma clones were then screened by indirect enzyme-linked immunosorbent assays (ELISA) for mAbs exhibiting strong reactivity to the P61 protein. Selected clones that produced mAbs against the P61 protein were subcloned three times by limiting dilution. Ascites was induced in pristine-primed BALB/c mice. Finally, the mAbs were identified by Western blot and indirect ELISA analyses. Additionally, mAb subtypes were identified using a SBA Clonotyping System/HRP Kit (Southern Biotech, USA).

### Expression of P61-derived polypeptides and screening by western blot analysis

To identify the approximate locations of the epitopes on the P61 protein, consecutively truncated P61 protein fragments were expressed in *E. Coli*. Primers were then designed that were specific for the P2–P19 fragments (Table 1). The P2, P3, and P4 fragment genes were cloned into pET30a vectors and the rest of the fragments were cloned into pMAL-c5x vectors. Recombinant BL21 *E. coli* cells were cultured at 37°C in LB medium containing 50 μg/ml kanamycin with agitation until their optical density (measured at 600 nm) reached 0.8. The cells were subsequently induced with 1 mM IPTG at 16°C for 6 h. Fragments that reacted with the mAbs were then screened by Western blot analysis. Briefly, the expressed polypeptides were subjected to SDS-PAGE and electro-blotted onto PVDF membranes. The membranes were then incubated with mAbs followed by a secondary HRP-conjugated goat anti-mouse antibody.

**Table 1:** Primers used for PCR amplification of truncated and overlapping fragments of the P61gene.

**Number**	**Peptides**	**Primer sequence (5′ to 3′)**	**Restriction enzyme**
1	P2	F CTACATATGACCAAGCCGACC	*NdeI*
		R CTAGGAATTCATGTGCAGATT	*EcoRI*
2	P3	F CTACATATGATGCAGTGGGAC	*NdeI*
		R CATGGATCCGTGGGCATCCGCATA	*BamHI*
3	P4	F CTACATATGAAGATGCTGCTG	*NdeI*
		R CTAAGAATTCACCCTGCGCCG	*EcoRI*
4	P5	F CTACATATGATGCAGTGGGAC	*NdeI*
		R CTAGTCGACTTAAACGTGCAGGGT	*SalI*
5	P6	F CTACATATGCGTAAAGACCTGTGGGA	*NdeI*
		R CTAGTCGACTTAGTGGGCATCCGCATAA	*SalI*
6	P7	F CTACATATGATGCAGTGGGACTTCTGGA	*NdeI*
		R CTAGTCGACTTAGTGCAGGGTCCAGCTTGGG	*SalI*
7	P8	F CTACATATGCACCGTAAAGACCTGTGGG	*NdeI*
		R CTAGTCGACTTAATCCGCATAAGCAAACACA	*SalI*
8	P9	F CTACATATGCCGGGTATTGGCTACTCCC	*NdeI*
		R CTAGTCGACTTACGCGTGTTCAATATAACCG	*SalI*
9	P10	F CTACATATGCCGGGTATTGGCTACTCC	*NdeI*
		R CTAGTCGACTTATGCACGCGGCAGGTTCGGC	*SalI*
10	P11	F CTACATATGGCCCACCGTTATCGTATTG	*NdeI*
		R CTAGTCGACTTACGGGGCCTGAGCCGGATCC	*SalI*
11	P12	F CTACATATGGTTCCGGTTTATGCGCCGA	*NdeI*
		R CTAGTCGACTTACGCGTGTTCAATATAACCG	*SalI*
12	P13	F CTACATATGGATGGTGGTCTGTGGGAAT	*NdeI*
		R CTAGTCGACTTAACCCTGCGCCGGAGGCGGG	*SalI*
13	P14	F CTACATATGGATGGTGGTCTGTGGGAAT	*NdeI*
		R CTAGTCGACTTACTGCGCATCATTAAAAACT	*SalI*
14	P15	F CTACATATGGAAGATGGCGATTTTACCC	*NdeI*
		R CTAGTCGACTTATTCAATGCGTTTACCAATT	*SalI*
15	P16	F CTACATATGCCGGTACTGTCCCGCGTAT	*NdeI*
		R CTAGTCGACTTAACCCTGCGCCGGAGGCGGG	*SalI*
16	P17	F CTACATATGCCGGTACTGTCCCGCGTAT	*NdeI*
		R CTAGTCGACTTAATCCAGACCCGCGCGAACG	*SalI*
17	P18	F CTACATATGACTAAAGTTGATCCAGAAA	*NdeI*
		R CTAGTCGACTTAACCCTGCGCCGGAGGCGGG	*SalI*
18	P19	F CTACATATGGAAGATGGCGATTTTACCC	*NdeI*
		R CTAGTCGACTTAATCCAGACCCGCGCGAACG	*SalI*
19	B	F CTACATATGCTGGTTCGTGAAGTTTTTAATG	*NdeI*
		R CTAGTCGACTTAATCCAGACCCGCGCGAACG	*SalI*
20	B1	F CTACATATGCTGGTTCGTGAAGTTTTTAATG	*NdeI*
		R CTAGTCGACTTAACCCGCGCGAACGCCTTC	*SalI*
21	B2	F CTACATATGCTGGTTCGTGAAGTTTTTAATG	*NdeI*
		R CTAGTCGACTTAGCGAACGCCTTCTTC	*SalI*
22	B3	F CTACATATGCGTGAAGTTTTTAATGATGC	*NdeI*
		R CTAGTCGACTTAACCCGCGCGAACGCCTTC	*SalI*
23	B4	F CTACATATGGTTTTTAATGATGCGCAGC	*NdeI*
		R CTAGTCGACTTAACCCGCGCGAACGCCTTC	*SalI*
24	B5	F CTACATATGAATGATGCGCAGCGCGATC	*NdeI*
		R CTAGTCGACTTAACCCGCGCGAACGCCTTC	*SalI*
25	B6	F CTACATATGCGCGATCGCCTGGTTTCTAAT	*NdeI*
		R CTAGTCGACTTAATCCAGACCCGCGCGAACG	*SalI*
26	B7	F CTACATATGCACGTTCTGGGCGGCGTTC	*NdeI*
		R CTAGTCGACTTAATCCAGACCCGCGCGAACG	*SalI*
27	B8	F CTACATATGCTGGGCGGCGTTCAGGAAC	*NdeI*
		R CTAGTCGACTTAACCCGCGCGAACGCCTTC	*SalI*
28	B9	F CTACATATGCTGGGCGGCGTTCAGGAAC	*NdeI*
		R CTAGTCGACTTACGCGCGAACGCCTTC	*SalI*

### Identification of the precise location of epitopes

In order to identify the minimal antigenic epitopes that were recognized by our P61 mAbs, consecutive primers (Table 1) or genes (Table 2) were synthesized and cloned into the pMAL-c5x expression vector. Maltose-binding protein (MBP)-fused recombinant proteins were expressed in BL21 *E. coli* cells and then identified by SDS-PAGE. These proteins were then used to determine the epitopes that were recognized by mAbs using the Western blot protocol described above.

**Table 2:** Gene sequences for cloning and expression of truncated peptides.

**Peptide**		**Gene sequence**
A1	F	TATGCTGTCCCGCGTATTTGAATATTGGACTAAAGTTGATCCAGAAATTGGTAAACGCATTGAAGAAGGCGTTCGCGCGGGTCTGGATTAAG
	R	TATGCTGTCCCGCGTATTTGAATATTGGACTAAAGTTGATCCAGAAATTGGTAAACGCATTGAAGAAGGCGTTCGCGCGGGTCTGGATTAAG
A2	F	TATGCGCGTATTTGAATATTGGACTAAAGTTGATCCAGAAATTGGTAAACGCATTGAAGAAGGCGTTCGCGCGGGTCTGGATTAAG
	R	AATTCTTAATCCAGACCCGCGCGAACGCCTTCTTCAATGCGTTTACCAATTTCTGGATCAACTTTAGTCCAATATTCAAATACGCGCA
A3	F	TATGTTTGAATATTGGACTAAAGTTGATCCAGAAATTGGTAAACGCATTGAAGAAGGCGTTCGCGCGGGTCTGGATTAAG
	R	AATTCTTAATCCAGACCCGCGCGAACGCCTTCTTCAATGCGTTTACCAATTTCTGGATCAACTTTAGTCCAATATTCAAACA
A4	F	TATGCCGGTACTGTCCCGCGTATTTGAATATTGGACTAAAGTTGATCCAGAAATTGGTAAACGCATTGAAGAAGGCGTTCGCGCGGGTTAAG
	R	AATTCTTAACCCGCGCGAACGCCTTCTTCAATGCGTTTACCAATTTCTGGATCAACTTTAGTCCAATATTCAAATACGCGGGACAGTACCGGCA
A5	F	TATGCCGGTACTGTCCCGCGTATTTGAATATTGGACTAAAGTTGATCCAGAAATTGGTAAACGCATTGAAGAAGGCGTTCGCTAAG
	R	AATTCTTAGCGAACGCCTTCTTCAATGCGTTTACCAATTTCTGGATCAACTTTAGTCCAATATTCAAATACGCGGGACAGTACCGGCA
A6	F	TATGCCGGTACTGTCCCGCGTATTTGAATATTGGACTAAAGTTGATCCAGAAATTGGTAAACGCATTGAAGAAGGCTAAG
	R	AATTCTTAGCCTTCTTCAATGCGTTTACCAATTTCTGGATCAACTTTAGTCCAATATTCAAATACGCGGGACAGTACCGGCA
A7	F	TATGGAATATTGGACTAAAGTTGATCCAGAAATTGGTAAACGCATTGAAGAAGGCTAAG
	R	AATTCTTAGCCTTCTTCAATGCGTTTACCAATTTCTGGATCAACTTTAGTCCAATATTCCA
A8	F	TATGTATTGGACTAAAGTTGATCCAGAAATTGGTAAACGCATTGAAGAAGGCTAAG
	R	AATTCTTAGCCTTCTTCAATGCGTTTACCAATTTCTGGATCAACTTTAGTCCAATACA
A9	F	TATGTGGACTAAAGTTGATCCAGAAATTGGTAAACGCATTGAAGAAGGCTAAG
	R	AATTCTTAGCCTTCTTCAATGCGTTTACCAATTTCTGGATCAACTTTAGTCCACA
A10	F	TATGACTAAAGTTGATCCAGAAATTGGTAAACGCATTGAAGAAGGCTAAG
	R	AATTCTTAGCCTTCTTCAATGCGTTTACCAATTTCTGGATCAACTTTAGTCA
A11	F	TATGTTTGAATATTGGACTAAAGTTGATCCAGAAATTGGTAAACGCATTGAAGAATAAG
	R	AATTCTTATTCTTCAATGCGTTTACCAATTTCTGGATCAACTTTAGTCCAATATTCAAACA
A12	F	TATGTTTGAATATTGGACTAAAGTTGATCCAGAAATTGGTAAACGCATTGAATAAG
	R	AATTCTTATTCAATGCGTTTACCAATTTCTGGATCAACTTTAGTCCAATATTCAAACA
A13	F	TATGTTTGAATATTGGACTAAAGTTGATCCAGAAATTGGTAAACGCATTTAAG
	R	AATTCTTAAATGCGTTTACCAATTTCTGGATCAACTTTAGTCCAATATTCAAACA
A14	F	TATGTTTGAATATTGGACTAAAGTTGATCCAGAAATTGGTAAACGCTAAG
	R	AATTCTTAGCGTTTACCAATTTCTGGATCAACTTTAGTCCAATATTCAAACA

### Reactivity of the identified B-cell epitopes with *N. brasiliensis*-positive mouse serum

To assess whether the screened B-cell epitopes of P61 could be identified by *N. brasiliensis*-positive mouse serum, the MBP-fused proteins containing the peptides A3, A7, B, B1, B6, and B7 were tested with *N. brasiliensis*-positive mouse serum using Western blot analysis, as described above. *N. brasiliensis*-positive mouse serum was used as the primary antibody and HRP-conjugated goat anti-mouse IgG was used as the secondary antibody.

### Location of the B-cell epitopes in the P61 protein

The three-dimensional (3D) structure of the P61 protein was unavailable in the Protein Data Bank (PDB). Consequently, we predicted its structure by 3D-modeling the P61 protein with the SWISSMODEL online sever. We then investigated the location of the epitopes on the protein using the Pymol software package, and presented them diagrammatically in the predicted protein model.

## Results

### Expression, purification, and identification of recombinant P61 protein

**Figure 1:**
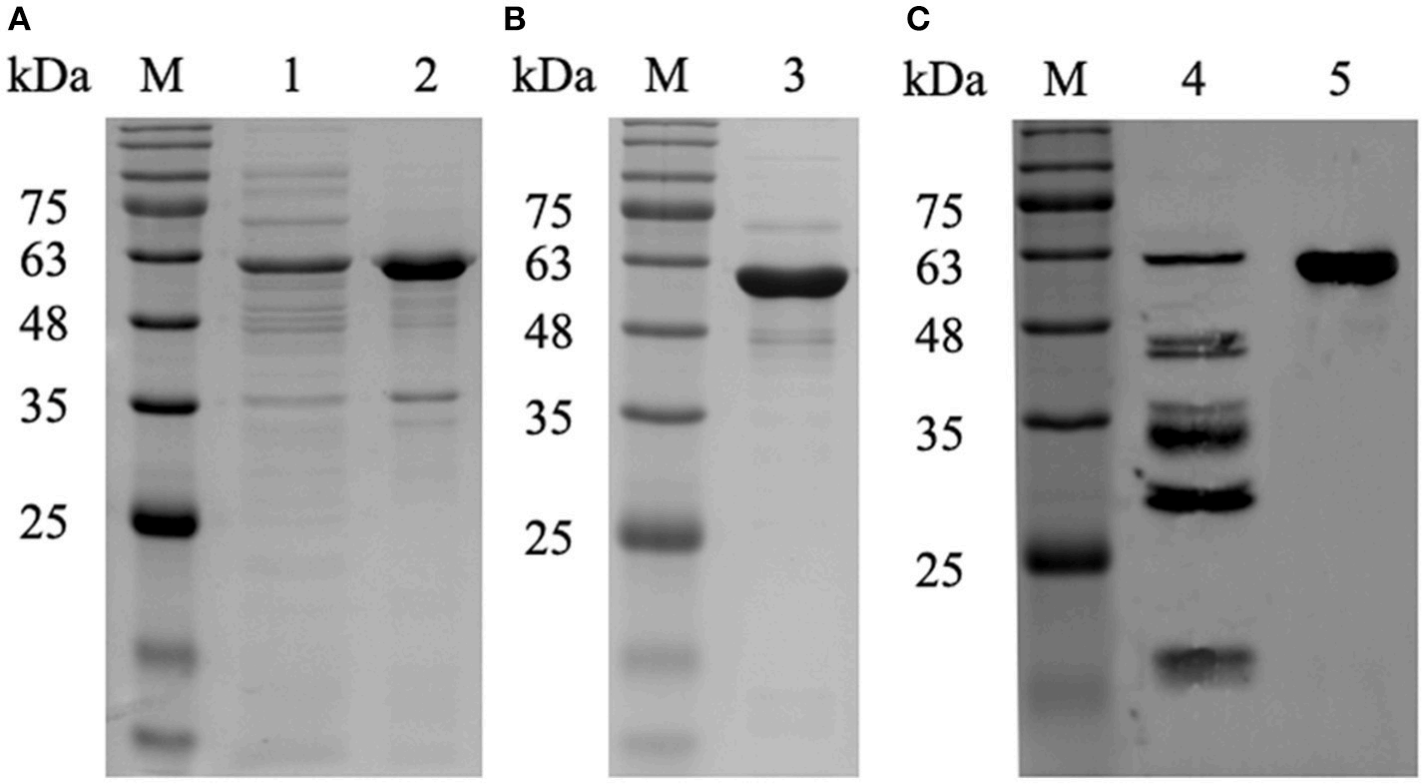
SDS-PAGE and Western blot analysis of the P61 protein. Lane M: protein marker. **(A)** Lanes 1 and 2: supernatant and precipitate fractions, respectively, of recombinant BL21 cells expressing P61 protein that were cultured for 6 h with 0.2 mM IPTG at 30°C. **(B)** Lane 3: purified P61 protein. **(C)** Lane 4: whole-cell protein of *N. brasiliensis* immunoblotted with the sera of mice infected with *N. brasiliensis*; Lane 5: purified P61 immunoblotted with the sera of mice infected with *N. brasiliensis*.

Recombinant P61 fused with a six-histidine tag (His-P61) was successfully expressed in BL21 (DE3) *E. coli* cells and a Ni–NTA affinity kit was used to produce purified P61 protein. Subsequent SDS-PAGE indicated that the P61 protein was soluble at 30°C, was present in its intact form, and had a molecular weight of 61 kDa (Figure 1A). Purified P61 protein exhibited a single band in Western blot analysis (Figure 1B). Western blot analysis further revealed that the P61 protein can be specifically identified by the endogenous antibodies present in serum from *N. brasiliensis*-infected mice (Figure 1C).

### Production and characterization of mAbs against P61 protein

**Figure 2:**
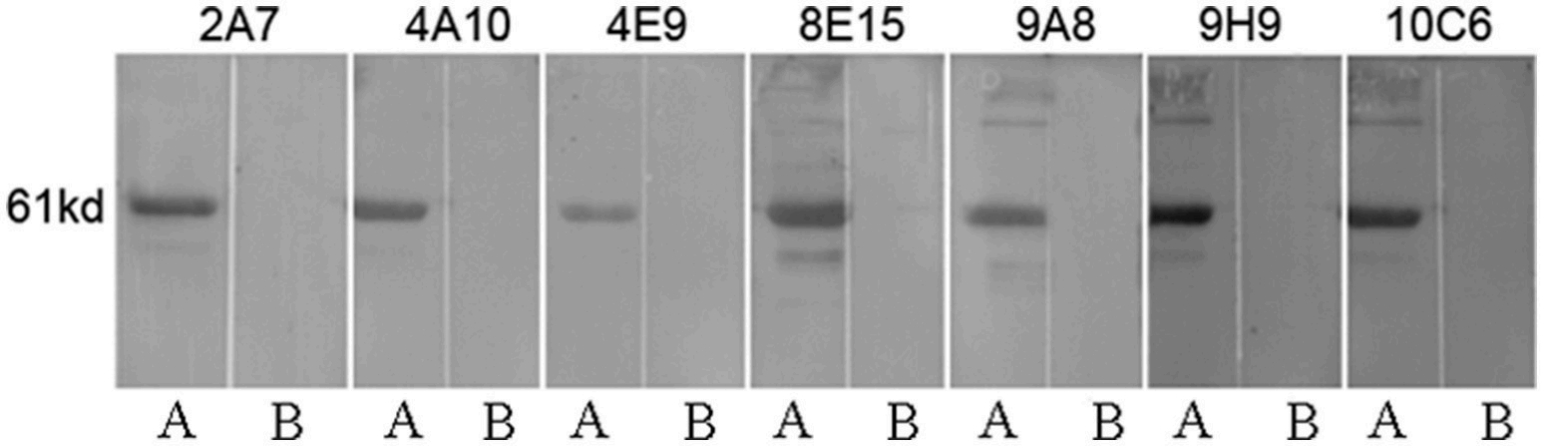
Specificity of the mAbs to recombinant His-P61 protein demonstrated by Western blot. Lane A: purified recombinant P61 protein; Lane B: recombinant His-Mce1C protein as the negative control.

After screening of hybridoma cells by indirect ELISA, we selected hybridoma cells that secreted antibodies to the P61 protein. Seven mAbs were thus generated and designated as 2A7, 4A10, 4E9, 8E15, 9A8, 9H9, and 10C6. The isotypes of the mAbs are shown in Table 3. The titers of the mAbs in cell cultures were greater than 1:2430 and the titers of the mAbs in the ascites fluid were 1:1 × 10^5^-1 × 10^6^. The mAbs reacted with recombinant P61 protein, but could not recognize another recombinant Mce1C protein that also contained the His fusion tag and that is also expressed by *Nocardia* spp. (Figure 2). These results indicate the specificity of the mAbs for the P61 protein of *N. brasiliensis*.

**Table 3:** The subtypes of the P61 mAbs.

**mAbs**	**2A7**	**4A10**	**4E9**	**8E15**	**9A8**	**9H9**	**10C6**
Heavy chain	IgG1	IgG1	IgG1	IgG1	IgG1	IgG1	IgG2b
Light chain	κ	κ	κ	κ	κ	κ	κ

### Identification of the truncated P61 protein

**Figure 3:**
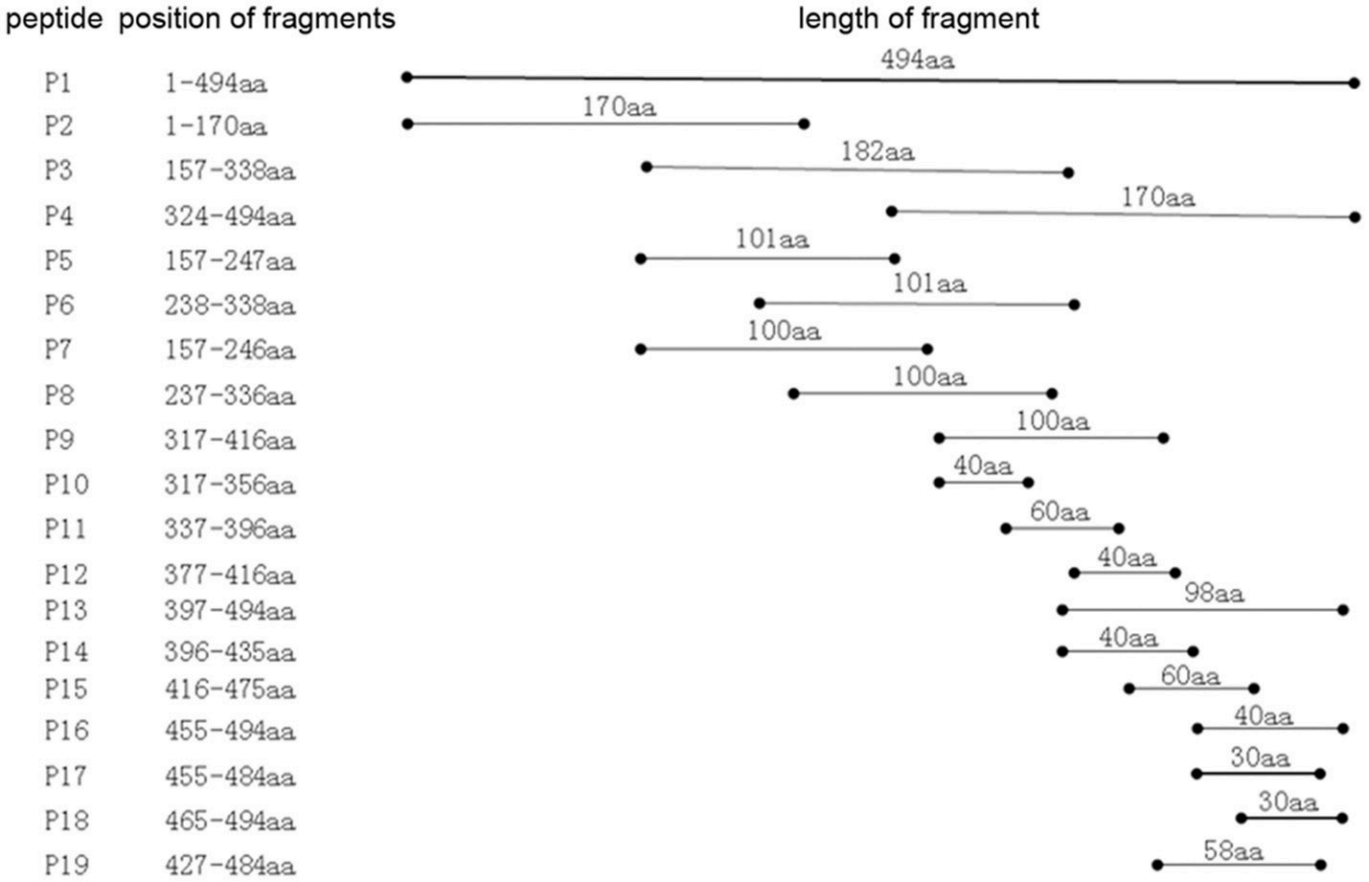
Schematic diagram of the P61 protein. Truncated peptides of the P61 protein were generated and used to identify P61 regions of reactivity with mAbs by Western blot analysis.

To identify the approximate location of the P61 epitope, a series of overlapping P61 peptides (designated P1–P19) were generated, expressed, and analyzed (Figure 3). Of these, mAb 8E15 recognized the P17 peptide of the P61 protein and mAbs 2A7, 4A10, 4E9, 9A8, 9H9, and 10C6 recognized the P19 peptide.

### Mapping of the epitopes to the P61 protein

**Figure 4:**
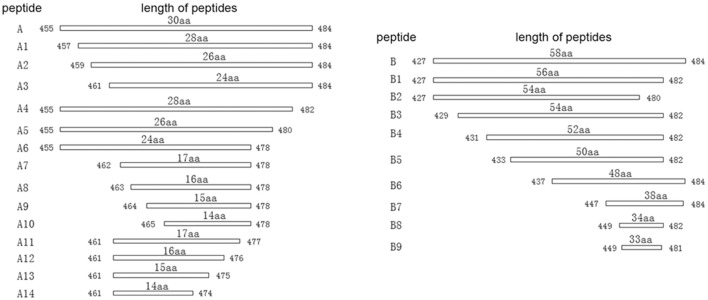
Schematic diagram of P17 (A) and P19 (B) fragments. The P17 (A) fragment was truncated into 14 different peptides for subsequent reaction with mAb 8E15 by Western blot analysis (left). The P19 (B) fragment was truncated into nine different peptides for subsequent reaction with mAbs 2A7, 4A10, 4E9, 9A8, 9H9, and 10C6 via Western blot analysis.

**Figure 5:**
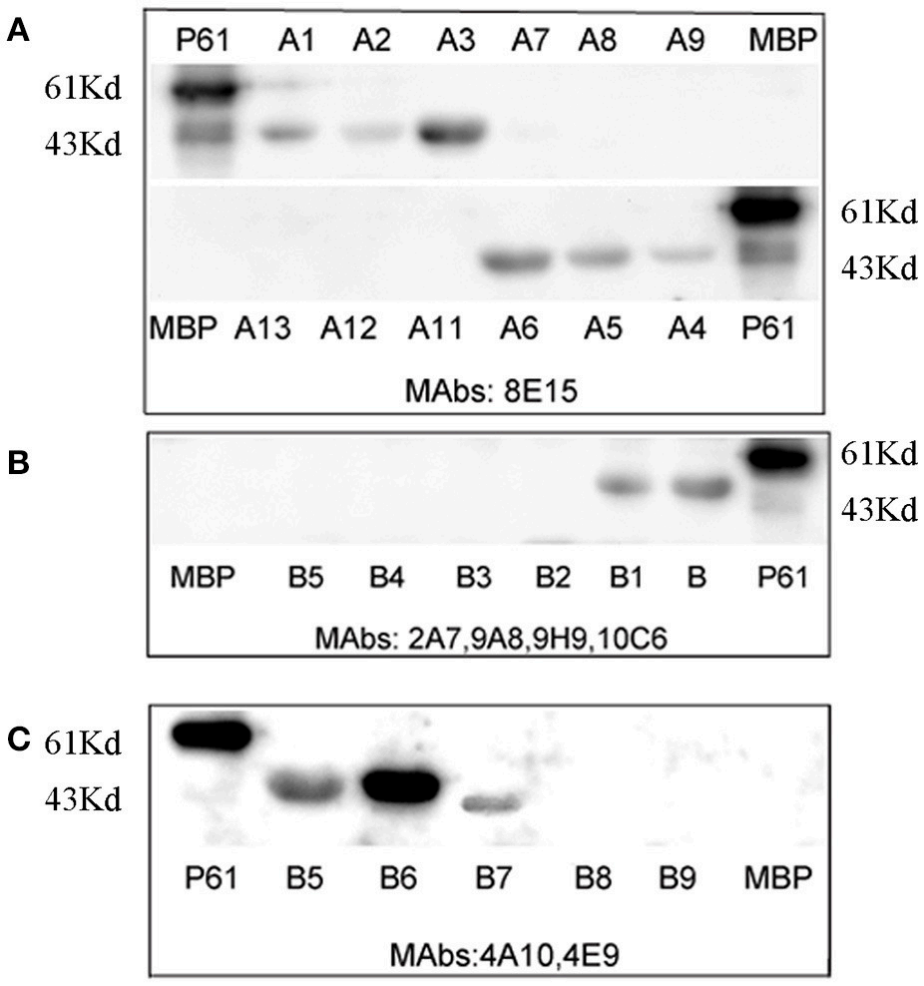
Minimal sequences of the epitopes were mapped using Western blot analysis. The full-length P61 protein was used as a positive control and MBP alone was the negative control in each blot. **(A)** The epitopes that were detected using mAb 8E15. **(B)** The epitopes detected using mAbs 2A7, 9A8, 9H9, and 10C6. **(C)** The epitopes detected using mAbs 4A10 and 4E9.

To map the minimal sequences of the epitopes recognized by the mAbs on the P61 protein, we generated a series of further truncated peptides from the P17 and P19 fragments (labeled A and B in Figure 4). These truncated peptides (A1–14 and B1–9) were reacted with the mAbs using Western blot analyses (Figure 5). Of these, mAb 8E15 reacted with fragments A1–A6, but not A7–A14; mAbs 4A10 and 4E9 reacted with fragments B5, B6, and B7, but not B8 and B9; and lastly, mAbs 2A7, 9A8, 9H9, and 10C6 reacted with fragments B and B1, but not B2–B5. Specifically, mAb 8E15 recognized the epitope comprising amino

acid (aa) positions 461–478 aa of the P61 protein, mAbs 4A10, and 4E9 recognized the epitope at 447–484 aa, and mAbs 2A7, 9A8, 9H9, and 10C6 recognized the epitope at 427–482 aa. All of the epitopes that were recognized by the antibodies were in overlapping regions of the P61 protein between amino acids 427 and 484.

### Reactivity of the identified B-cell epitopes with *N. brasiliensis*-positive mouse serum

**Figure 6:**
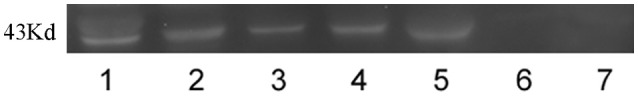
Reactivity of epitopes was analyzed with *N. brasiliensis*-positive mouse serum by Western blot. Lane 1–6: peptide 427–484 aa, peptide 427–482 aa, peptide 437–484 aa, peptide 447–484 aa, peptide 461–484 aa, and peptide 462–478 aa, respectively, immunoblotted with *N. brasiliensis*-infected mouse serum. Lane 7: MBP (negative control).

The antigenicities of the screened epitopes were determined with *N. brasiliensis*-positive mouse serum. Peptides of P61 encompassing the regions 427–484, 427–482, 437–484, 447–484, and 461–484 aa reacted with *N. brasiliensis*-positive mouse serum (Figure 6). The peptide within the amino acid sequence 461–478 aa did not react at all with the infected serum. These results suggest that the screened epitopes at positions 427–482 and 447–484 aa are robustly identified by *N. brasiliensis*-positive mouse serum, and can potentially be used as markers for the diagnosis of nocardiosis caused by *N. brasiliensis* infection.

### Location of the screened epitopes in the fully folded P61 protein

**Figure 7:**
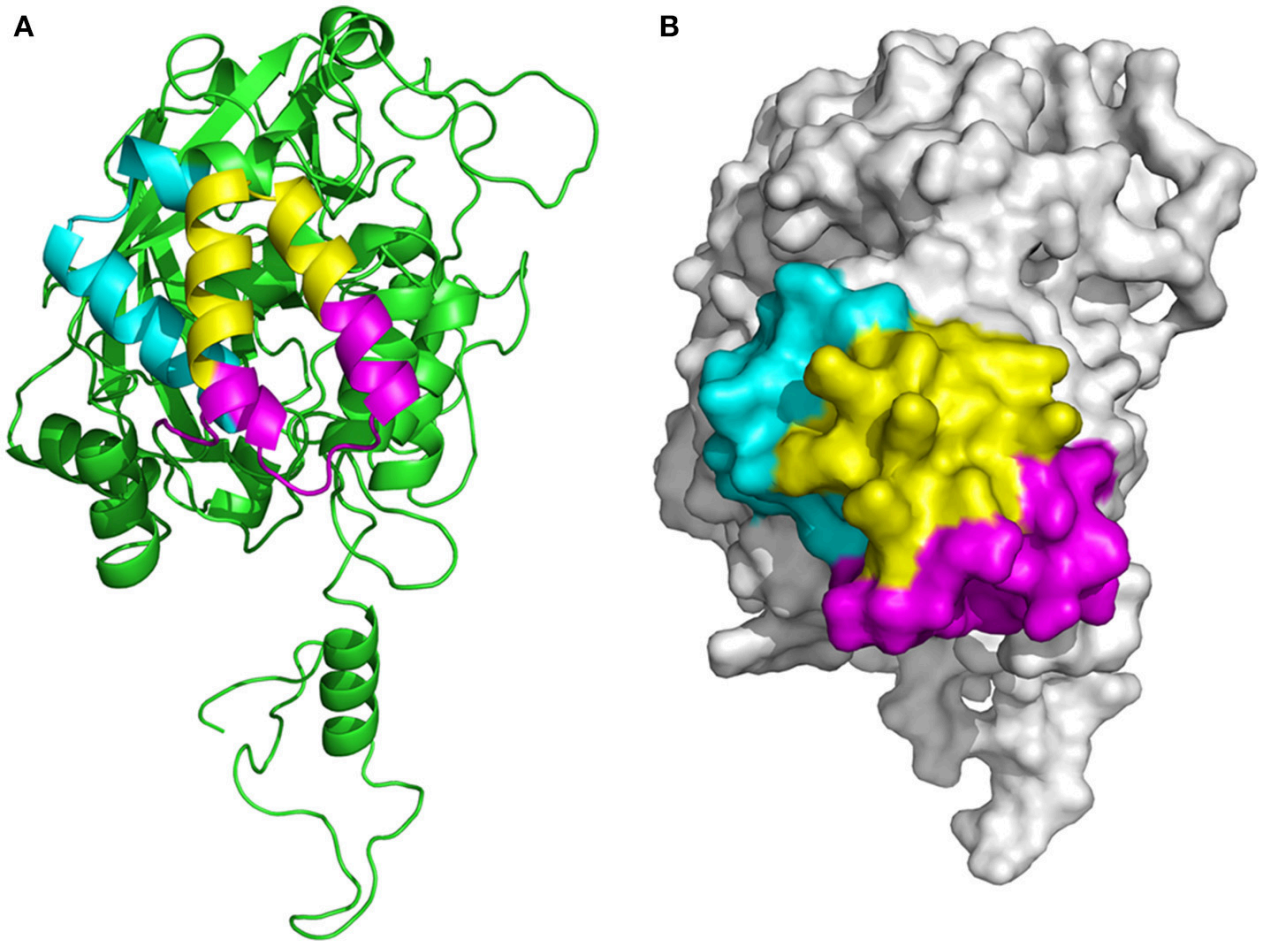
Determination of the location of epitopes on the P61 protein. **(A)** Ribbon diagram of the P61 protein. **(B)** Space-filling 3D model of P61. The screened epitopes (of P61 amino acid sequences) 427–446 aa is highlighted in cyan, 447–460 and 479–484 aa are highlighted in magenta, and 461–478 aa is highlighted in yellow.

The location of the screened, overlapping epitopes comprising the positions 427–482, 447–484, and 461–478 aa were determined within the three–dimensional structure of the P61 protein. The 3D structural modeling analyses indicated that these three peptides were located on the external surface of the P61 protein (Figure 7).

## Discussion

The P61 protein is a highly conserved, immunodominant protein in *Nocardia* bacteria, and is the target of the body's humoral response to *Nocardia* infection. Thus, the protein is a potentially valuable tool for nocardiosis disease diagnosis (Vera-Cabrera et al., 1999). Peptide-based tools play a critical role in clinical diagnoses and the use of a specific epitope to diagnose nocardiosis could avoid misdiagnoses that result from cross-reaction with bacteria similar to *N. brasiliensis*. However, no studies have been conducted to investigate epitopes of the *N. brasiliensis* P61 protein. In this study, mice were immunized with purified, recombinant His-P61 protein and seven mAbs were produced and were then used to screen for the B-cell epitope of P61. All of the mAbs reacted with the full-length P61 protein and truncated peptides of P61.

During initial screening, the P61 peptide comprising the amino acid sequence positions of 455–484 aa was screened by mAb 8E15 and the peptide comprising the positions 427–484 aa was screened by mAbs 2A7, 4A10, 4E9, 9A8, 9H9, and 10C6. These results indicated that the amino acid sequences recognized by the mAbs clearly overlap. As expected, mAb 8E15 recognized both of the peptides, while mAbs 2A7, 4A10, 4E9, 9A8, 9H9, and 10C6 only reacted with the peptide comprising positions 427–484 aa. To identify the minimal epitope (minimum amino acid positions of P61 required for antibody binding), a series of further truncated peptides were reacted with the same mAbs by Western blot analysis. Three minimal sequences were subsequently isolated: the epitope 461-FEYWTKVD PEIGKRIEEG-478 was recognized by mAb 8E15; the epitope 427-LVREVFNDAQRDRLVSNVVG GVQEPVLSRVFEYWTKV.

DPEIGKRIEEGVRAG-482 was recognized by mAbs 2A7, 9A8, 9H9, and 10C6; and the epitope 447-HVLGGVQEP VLSRVFEYW TKVDPEI GKRIEE GVRAGLD −484 was recognized by mAbs 4A10 and 4E9. These three epitopes were then expressed in the pMAL-c5x plasmid vector and their reactivity with *N. brasiliensis*-positive mouse serum was determined. Results from these experiments indicated that epitopes comprising the positions 427–482 and 447–484 aa were identified by *N. brasiliensis*-positive serum, but that epitope 461–478 aa was not identified by the serum. Interestingly, the peptide comprising positions 461–484 aa, which overlapped with the 461–478 aa peptide, successfully reacted with infected mouse serum, suggesting that positions 461–484 on the P61 protein contains critical binding sites for the mAbs. However, it is possible that the MBP fusion tag was large enough to block the binding sites in peptide 461–478 aa, while leaving sufficient binding sites open on the larger peptide 461–484 aa. The SWISSMODEL online server was used to predict the 3D structure of the P61 protein and Pymol was used to map the locations of the screened epitopes on the 3D model of P61.

All three screened peptides were identified on the external surface of the P61 protein, indicating that they would be easily recognized by the infected host's immune cells. In future investigations, serum will be collected from patients infected with *N. brasiliensis* to verify the applicability of the screened peptides for clinical diagnosis of this bacterial infection. Peptides will then be synthesized and their reactivity will be verified using ELISA.

In conclusion, we produced seven mAbs for the P61 protein of *N. brasiliensis* and isolated three epitopes from the P61 protein (461–478, 427–482, and 447–484 aa) that can facilitate the future development of a serodiagnostic tool for nocardiosis diagnosis.

## Author contributions

XJ and JL conceived and designed the experiments. XJ wrote the manuscript. XJ and NS performed the experiments. XJ and XH analyzed the data. QL, SX, TQ, LT, and BW contributed reagents, materials, analysis tools. JL supported the research financially and administratively, and provided final approval of manuscript.

### Conflict of interest statement

The authors declare that the research was conducted in the absence of any commercial or financial relationships that could be construed as a potential conflict of interest.
